# In vivo evaluation of a cytochrome P450 gene from poultry red mite, *Dermanyssus gallinae*, as a vaccine antigen for chicks

**DOI:** 10.1186/s13071-025-07218-8

**Published:** 2026-02-09

**Authors:** Jing Liu, Meng Wu, Shuo Yin, Zhonghao Wang, Zhengjie Wang, Junlong Liu, Jianhua Qin, Yicun Guo, Jianhua Zhang, Chuanwen Wang, Yuzhu Zuo

**Affiliations:** 1 College of Veterinary Medicine, Hebei Agricultural University, Baoding, 071001 Hebei China; 2 Shijiazhuang Rongchuan Feed Co., Ltd, Shijiazhuang, 051430 Hebei China

**Keywords:** *Dermanyssus gallinae*, Cytochrome P450, Vaccine candidate, *Deg-CYP-3*

## Abstract

**Background:**

*Dermanyssus gallinae* is a prevalent ectoparasite in the poultry farms, inflicting damage on chicken health through blood-sucking. Chemical acaricides commonly used for mite control often show reduced efficacy due to the development of resistance. Therefore, alternative control methods are needed, and vaccination is a promising strategy for controlling *D. gallinae*.

**Methods:**

The mRNA expression of *Deg-CYP-3* in mites at various developmental stages, as well as under fed and starved conditions, was analyzed. Subsequently, recombinant protein rCYP-3 was induced, purified, and employed for immunization. Following immunization, antibodies were analyzed and mite challenge was then conducted. Following a 12 h period of blood-feeding on chicks, the mites were collected to evaluate the acaricidal efficacy of the rCYP-3 vaccine.

**Results:**

The *Deg-CYP-3* gene was expressed across all life stages and maintained stable expression levels under both fed and starved conditions. The recombinant protein rCYP-3 was successfully expressed in *Escherichia coli* and efficiently secreted into the culture supernatant. Immunization with rCYP-3 induced a specific IgY immune response in chicks, as confirmed by ELISA. Moreover, anti-rCYP-3 serum specifically recognized P450 proteins extracted from *D. gallinae*, as demonstrated by Western blot analysis. Immunization resulted in an 8.1% reduction in adult mite survival (*P* > 0.05), whereas nymph survival decreased significantly by 22.4% (*P* < 0.01). In addition, oviposition rate, hatching rate, and fecundity were reduced by 2.8%, 2.2%, and 22.0%, respectively, in the immunized group. Overall, vaccine efficacy was calculated to be 30.6% in immunized birds. Furthermore, the expression level of Deg-CYP-3 in mites fed on immunized hosts was significantly lower than that in mites from the unimmunized control group.

**Conclusions:**

Our findings demonstrated that the *Deg-CYP-3* gene exhibits high transcriptional activity during both the adult and nymph stages of *D. gallinae*. Moreover, its expression remains consistent regardless of the feeding status of adult mites. Immunization with rCYP-3 effectively reduced mite survival, reproductive capacity, and gene expression levels, demonstrating its potential as a preventive and control strategy against *D. gallinae*.

**Graphical Abstract:**

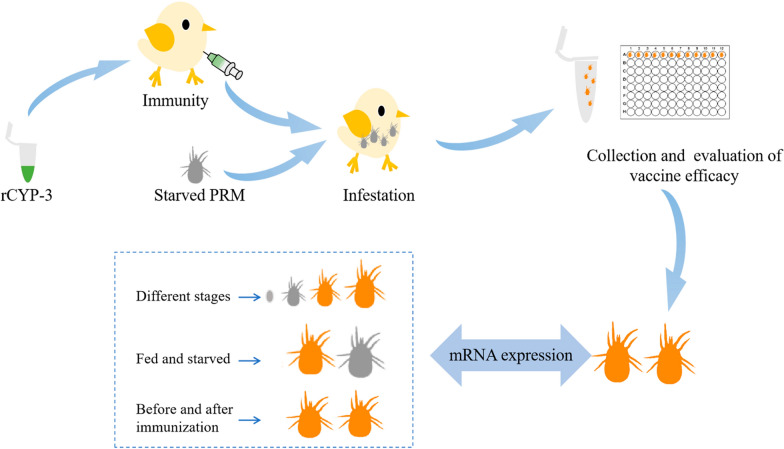

**Supplementary Information:**

The online version contains supplementary material available at 10.1186/s13071-025-07218-8.

## Background

Poultry red mites (*Dermanyssus gallinae*, PRM) are one of the most harmful ectoparasites of the avian species, including chickens [1]. On average, 83.0% of farms in 11 European countries have been reported to be infested with *D. gallinae* [2]. In Poland and Slovakia, for instance, the infestation rate has been recorded at up to 100.0% in certain regions [3]. In addition, mite infections have been documented in a number of other countries, including Japan, Australia, and Brazil. The infection of poultry by *D. gallinae* can lead to anemia, gradual feather loss, itching, weight loss, and stress symptoms, resulting in decreased quality and quantity of eggs [4–6]. The hypothesis has been put forward that the *D. gallinae* might act as a vector for various pathogens, including the avipox virus, the avian influenza virus, *Salmonella* Gallinarum, and *Erysipelothrix rhusiopathiae* [1, 7, 8]*.* It is also reported that *D. gallinae* infestations caused dermatitis in poultry workers and residents nearby poultry houses [9]. Therefore, an alternative method of controlling *D. gallinae* needs to be urgently established.

Spraying chemical acaricides on cages and houses is the most common method of controlling *D. gallinae*. However, in most cases, the behavior of *D. gallinae* hiding in cracks and crevices makes control ineffective [10]. Incomplete and long-term use of pesticides (e.g. organophosphates, organochlorines, carbamates, amitraz and pyrethroids) also induce heritable resistance in mites, undesirable effects on nontarget organisms, threats to animal and human health, and environmental problems [11]. Therefore, other management strategies are required to ensure animal welfare and reduce economic losses in poultry farming [2, 10]. Alternative strategies for controlling *D. gallinae* have been studied and proposed, including developing repellents using plant essential oils [10, 11] and the implementation of vaccination in chickens [12, 13], with the aim of inducing protective effects against *D. gallinae*. Among these alternative strategies, there has been a focus on vaccination, and its notable benefits include the extension of the duration of efficacy, its environmental friendliness due to the absence of chemical residues, and its substantial reduction in the risk of resistance. It has been reported that antigens derived from *D. gallinae* include rDg-CatD-1, rDg-CatL-1, rDg-Lgm, Deg-CPR-1, and Dg-Cys [14–17]. However, the *D. gallinae* can evade the action of these antigens through the secretion of immunosuppressive molecules, thereby attenuating the vaccine-induced protective immune response. As a consequence, no commercial vaccines targeting *D. gallinae* have been implemented to date. To tackle this challenge, further investigations are required to identify additional potential candidate antigens for vaccine development.

*Cytochrome P450-dependent monooxygenases* (P450s) represent a pivotal metabolic system, given their role in regulating the levels of endogenous compounds, including hormones, fatty acids and steroids. In addition, they play a crucial part in the catabolism and anabolism of xenobiotics, such as drugs, pesticides, and plant toxins. These enzymes are found in virtually all aerobic organisms, including insects, plants, mammals, birds, and bacteria [17, 18]. To date, the identification of cytochrome P450 genes in insects has reached more than 1000, with the majority found in the microsomal CYP4, CYP6, CYP9, CYP28, CYP321, and mitochondrial CYP12 families [19]. Furthermore, a significant number of cytochrome P450 genes, particularly those belonging to the CYP4, CYP6, CYP9, and CYP12 families, have been shown to play a crucial role in insecticide metabolism and resistance [18]. As demonstrated in the research conducted by Joußen et al. [19] and Yang et al. [20], the presence of P450s has also been reported in *Drosophila melanogaster* and *Helicoverpa armigera*, respectively. Recently, four P450s genes of *D. gallinae* have been identified and then characterized by Wang et al. [21]. However, they did not continue to analyze the function of P450s as a vaccine antigen. As for the targets of vaccine antigens for the prevention of hematophagous ectoparasites, two types of antigens can be considered: the first group comprises secreted proteins from salivary glands, which facilitate attachment to host skin and blood-sucking by suppressing host immune responses. The other group includes antigens expressed in the midguts, as the ingested blood accumulates in these organs, and midgut antigens can be efficiently exposed to antibodies present in the blood ingested from immunized animals [22]. The P450s have been demonstrated to be expressed in the midgut, as exemplified by *D. melanogaster* and *H. armigera* [23, 24]. It is worth noting that in our previous experiments, the Deg-CYP-3 protein of *D. gallinae* has been confirmed to be present in the digestive tract and Malpighian tubules. Enzyme activity tests of recombinant Deg-CYP-3 protein have also been carried out, and rCYP-3 has been confirmed to be active against p-nitroanisole, which further validates its potential as a vaccine antigen for *D. gallinae* (unpublished). Therefore, the rCYP-3 protein may be suitable for development as a vaccine antigen against *D. gallinae*.

In this study, the *Deg-CYP-3 gene* of *D. gallinae* was selected to evaluate the potential for vaccine development, which plays an important role in the drug resistance and is distributed in the digestive tract and Malpighian tubules. To characterize the Deg-CYP-3 gene, the mRNA expression in six strains of *D. gallinae* at different developmental stages, as well as under fed and starved conditions was analyzed. Then, the rCYP-3 protein of *D. gallinae* was expressed in *Escherichia coli*. Chicks were immunized with rCYP-3 protein to produce specific antibodies, and the reactivity of immune serum against natural Deg-CYP-3 protein from *D. gallinae* was analyzed. Finally, the acaricidal effects of antibodies against the rCYP-3 on *D. gallinae* were evaluated using in vivo feeding assays. This study provides a basis for understanding the function of the *Deg-CYP-3* gene in mites as vaccine.

## Methods

### *Dermanyssus gallinae* used in the experiments

In this study, six populations of *D. gallinae* were used. Among them, four strains (HD, DK, CY, and BGY-5) were collected from Hebei Province, China in 2023, and two strains (SS and PG) were donated by Professor Pan Baoliang from the College of Veterinary Medicine, China Agricultural University. The SS strain was selected at random for the *Deg-CYP-3* gene cloning, recombinant protein expression and vaccine evaluation. In addition, the SS strain was utilized as a reference benchmark for the subsequent gene expression analysis with other strains.

All the strains of mites were reared on chicks and maintained under conditions at 30 °C, 75.0% relative humidity, and 12 h light/dark photoperiods. Chicks were housed in an experimental animal room, and water and feed were provided ad libitum. The experiments on animals were approved by the Institutional Animal Care and Use Committee of Hebei Agricultural University (approval no. 2024050).

### RNA isolation and cDNA synthesis

Total RNA was extracted from 50 adult female mites (SS strain) using TRIzol Reagent (Tiangen Biotech Co., Ltd., China), following the manufacturer’s instructions. First-strand cDNA was then synthesized from two mg RNA using EasyScript One-step gDNA Removal and cDNA Synthesis SuperMix (TransGen Biotech Co., Ltd., China). The synthesized cDNA was then used as the template for the PCR amplification of the full-length *Deg-CYP-3* gene.

### Amplification of the *Deg-CYP-3* gene

Primers for amplifying the *Deg-CYP-3* gene of *D. gallinae* were designed based on the reference sequence of *D. gallinae* (Accession No. MN695338). The open reading frames of *Deg-CYP-3* from the SS, DK, and HD strains were amplified by PCR using strain-specific primers (Table 1), which were designed and synthesized by Sangon Biotech Co., Ltd. (China). PCR amplifications were carried out in an Applied Biosystems Veriti Thermal Cycler (Thermo Fisher Scientific Co., Ltd., China). Each 20 μL reaction mixture contained 10 μL of 2 × PrimeSTAR Max DNA Polymerase (Vazyme Co., Ltd., China), 3 μL of cDNA template, 0.4 μL of forward primer (10 μM), 0.4 μL of reverse primer (10 μM), and 6.2 μL of ddH_2_O. The thermal cycling conditions consisted of an initial denaturation at 95 °C for 5 min, followed by 30 cycles of denaturation at 95 °C for 45 s, annealing at 55 °C for 2 min, extension at 72 °C for 1.5 min, and a final extension at 72 °C for 10 min. Following gel electrophoresis and purification, the PCR products were ligated into the pMD-19T Simple Cloning Vector and transformed into *E. coli* Trans-T1 competent cells. Positive clones were selected and sequenced (Sangon Biotech Co., Ltd., China) to confirm insert identity, and designated as pMD-19T-Deg-CYP-3.

**Table 1:** Primers used for PCR and qRT-PCR

Primer name	Sequence (5´ → 3´)	Application	Length (bp)
Deg-CYP-3-F	CCGGAATTCATGTGGGCCGTTCTAGTGTTA	PCR for protein expression	1485
Deg-CYP-3-R	CCGCTCGAGAGAAACATAGCTGGCTTT		
qCYP-3-F	GCATACTGGCTGAGAA	PCR and qRT-PCR	285
qCYP-3-R	CCTGATACTGGACATCGC		
Actin-F	CCCATGCTATCCTGCGT		169
Actin-R	GCCATTTCCTGCTCAAAG		

Restriction endonuclease sequences are highlighted in bold, while F and R mean forward and reverse primer, respectively

### Gene expression analysis of *Deg-CYP-3*

The mRNA expression of the *Deg-CYP-3* gene in six *D. gallinae* strains across different developmental stages-eggs, larvae, nymphs, and adults—and under both fed and starved conditions was analyzed using standard PCR. To assess the transcriptional level of *Deg-CYP-3* under nutritional stress, 50 engorged adults and 50 adults starved for 7 days from each strain were collected for total RNA extraction. To investigate developmental stage-specific expression patterns of *Deg-CYP-3*, 300 individuals each of eggs, larvae, nymphs, and adults from each strain were separately collected for RNA isolation. Total RNA extraction and cDNA synthesis were performed as previously described. RNA concentration was measured using a Nano-800 + Nucleic Acid and Protein Concentration Analyzer (Shanghai Jiapeng Technology Co., Ltd., China). During cDNA synthesis, equal amounts of total RNA were used across all reaction groups to ensure consistency. The primers used for PCR amplification are listed in Table 1. PCR was carried out with Ex-Taq polymerase (Tiangen Biotech Co., Ltd., China) in a 20 μL reaction volume according to the manufacturer’s instructions. Amplified products were separated by electrophoresis on a 1.0% agarose gel. The actin gene was used as an internal reference control, and distilled water was included as a negative control in place of cDNA template to monitor potential contamination.

Quantitative Real-Time PCR (qRT-PCR) was also utilized for the analysis of *Deg-CYP-3* gene expression levels at different developmental stages of eggs, larvae, nymphs, and adults in the SS strain, as well as under fed and starved conditions in six mite strains. The cDNA synthesized in the above-mentioned PCR experiment was used as the qRT-PCR template for the *Deg-CYP-3* gene. Table 1 shows the primers used for qRT-PCR. The 20 μL reaction mixtures contained 10 μL PerfectStart Green qPCR SuperMix (TransGen Biotech Co., Ltd., China), 7.2 μL ddH_2_O, 0.4 μL forward primer, 0.4 μL reverse primer, and 2 μL templates. The cycling conditions consisted of initial denaturation at 95 °C for 30 s, followed by 40 cycles at 95 °C for 5 s, 55 °C for 30 s, and 72 °C for 30 s. To evaluate the specificity of primer pairs, we performed a final melting curve analysis from 60 °C to 95 °C at a rate of 0.1 °C/s. The experiment was carried out at least in triplicate. The qRT-PCR was performed using the CFX Connect Real-Time PCR Detection System. The expression level of the *Deg-CYP-3* gene was analyzed using the 2^−ΔΔCt^ method [25].

### Preparation of recombinant *Deg-CYP-3* and western blot analysis

The recombinant plasmid of pMD-19T-Deg-CYP-3 of SS strain was digested with restriction endonuclease *EcoR*I and *Xho*I, and then cloned into the pET32a expression vector containing His-tagged coding sequence. The constructed recombinant plasmid was confirmed by sequencing, and named pET32a-Deg-CYP-3. Then, the constructed plasmid was transformed into *E. coli* Transetta (DE3) pLysS (TransGen Biotech Co., Ltd., China). The *E. coli* cells carrying pET32a-Deg-CYP-3 plasmid were incubated in Luria–Bertani (LB) medium overnight, after which the cultures were transferred to fresh LB medium. The induction of protein expression was achieved by the addition of a final concentration of 0.2 mM isopropyl-b-D-thiogalactopyranoside (IPTG) to the medium when the optical density at 600 nm (OD_600_) of the bacterial solution was approximately 0.5–0.6. Thereafter, the bacterial suspension was collected after a 24 h period and subjected to lysis by ultrasonication. Following the confirmation of recombinant protein expression by 10.0% SDS-PAGE, the purification process was then undertaken using a Ni–NTA spin column (TransGen Biotech Co., Ltd., China), and the resultant protein was subsequently dialyzed against phosphate buffered saline (PBS). The purity of the recombinant Deg-CYP-3 protein (rCYP-3) was evaluated by 10.0% sodium dodecyl sulfate–polyacrylamide gel electrophoresis (SDS-PAGE). The purified rCYP-3 protein was analyzed by SDS-PAGE and subsequently transferred onto a polyvinylidene difluoride (PVDF) membrane. Western blot (WB) analysis was conducted using a monoclonal anti-His antibody (Tiangen Biotechnology Co., Ltd., China) to verify the presence of the target protein. Protein concentration was measured using the BCA Protein Assay Kit (Beijing Solarbio Science & Technology Co., Ltd., China), with bovine serum albumin (BSA) as the standard, according to the manufacturer’s instructions.

### Immunization of chicks with rCYP-3

For immunization experiment, six 8-week-old healthy specific pathogen-free (SPF) chicks (White Leghorn, China) were selected and randomly allocated into two groups (group I and II), with three chicks per group. These chicks were housed individually. On day 0 (V1), chicks in group I (immunized group) received intramuscular injections of 100 μg rCYP-3 formulated in complete Freund’s adjuvant (Sigma, Missouri, USA) into the pectoral muscle, whereas those in group II (unimmunized group) were administered an equivalent volume of PBS formulated in complete Freund’s adjuvant. Subsequent booster immunizations were performed on days 14 (V_2_), 28 (V_3_), and 42 (V_4_), with group I receiving 50.0 μg rCYP-3 and group II receiving PBS, both formulated in incomplete Freund’s adjuvant (Sigma, Missouri, USA). The health status of all chicks was monitored twice daily throughout the experiment. For this experiment, two batches of independent experiments were conducted, with six chickens in each batch of experiment, indicating good repeatability.

### Polyclonal antibodies and western blot analysis

Chick serum containing specific anti-rCYP-3 polyclonal antibody was collected prior to each injection, and additionally, serum was collected 14 days after the final injection and stored at −20 °C for further analysis. The IgY antibody level in sera against rCYP-3 was determined by enzyme-linked immunosorbent assay (ELISA). Sera from unimmunized chicks were used as negative controls. ELISA plates were coated overnight at 4 °C with 0.2 μg/well of rCYP-3 protein. Following this, the plates were washed five times with 200 μL of PBST (PBS containing 0.05% v/v Tween-20), and then blocked with 300 μL/well of 5% skim milk for 2 h at 37 °C in an incubator. Following washing, a diluted serum sample (1:200) was added to the antigen-coated wells and incubated at 37 °C for 1.5 h. The bound antibodies were subsequently detected using goat anti-chicken IgG conjugated with HRP (1:5000) (Biosynthesis Biotechnology Co. Ltd., China). In order to visualize the reaction, 100 μL of TMB Single-Component Substrate solution (Beijing Solarbio Science & Technology Co., Ltd., China) was added. The reaction was then halted by the addition of 50 μL/well sulfuric acid, and the optical density (OD) was measured at 450 nm in a microplate reader (Bio-Rad Laboratories, California, USA). The ELISA results were analyzed in reference to Meng et al. [26].

Concurrently, the reactivity and specificity of chick serum were assessed using WB analysis. Five female mites were placed in a 1.5 mL Eppendorf tube containing 25 μL of PBS (0.04 M, pH 7.0). The mites were then homogenized thoroughly using a grinder (Tiangen Biotech Co., Ltd., China). Following homogenization, the mixture was centrifuged at 4 °C and 12,000 × *g* for 10 min, and the resulting supernatant was used as the crude protein source. The protein concentration in the *D. gallinae* homogenate was determined using the BCA protein assay kit. Subsequently, 20 μg of soluble mite crude protein extracts were separated by SDS-PAGE and transferred onto PVDF membranes. The chick serum was diluted at a ratio of 1:200, while the secondary antibody using goat anti-chicken IgG conjugated with HRP (1:8000). Finally, immunoreactive signals were visualized using an ECL Western Blotting Substrate (NCM Biotech Co., Ltd., China).

### Evaluation of vaccine efficacy

Vaccine efficacy was assessed using a method based on the in vivo rearing system developed by Wang et al. [27]. This system is composed of a metal cage, plastic storage box, and a water-filled tray. Vaseline is applied to the top edges of the plastic storage box, which is subsequently placed on the aforementioned water-filled tray, to prevent mites from escaping from the feeding system. On day 56, each chick was challenged with 400 adult females and 400 nymphs that had been starved for 3 days. Following a 12-h period of blood-feeding on chicks, over 300 engorged living adult females or nymphs were collected from the immunized and unimmunized groups. The recovery rates were generally consistent across two groups, and 300 mites were selected from each group for subsequent experiments. Three replicates were included in each group.

A total of engorged 50 adult females and 50 nymphs were selected at random and transferred into individual wells of a 96-well tissue culture plate, which were incubated at 30 °C with 75.0% relative humidity. Then, it was continuously observed under a stereomicroscope for 5 days. The following parameters were primarily observed and recorded: the mortality rate (Formula [1]), oviposition rate (Formula [2]), reduction on oviposition rate (Formula [3]), fecundity (Formula [4]), reduction on fecundity (Formula [5]), egg hatching rate (Formula [6]), and reduction on egg hatching rate (Formula [7]). Notably, an assessment of digestion rate of mites (Formula [8]), reduction on digestion rate (Formula [9]), success rate (Formula [10]), and vaccine efficacy (Formula [11]) of the adults was conducted in the experiment. Mite digestibility was specifically evaluated by selecting and weighing 50 adult mites starved for 3 days, subsequently selecting and immediately weighing engorged 50 adult mites collected after 12 h blood-feeding, and finally re-weighing the same 50 adult mites after an additional 3 days of digestion. The effects of immunization on *D. gallinae* were evaluated employing the following formulae [14, 28]:

The cumulative mite mortality rate (M):

1 $${\mathrm{M}} (\%)=\frac{\text{No. of dead mites}}{\text{No. of collected mites}}{\times}{100\%}$$

The oviposition rate of mites (O):

2 $${\mathrm{O}} (\%)=\frac{\text{No. of mites laying eggs}}{\text{No. of mites}}{\times}{100\%}$$

Reduction on oviposition rate of mites (RO):

3 $${\mathrm{RO}} (\%)=(1-\frac{\mathrm{Ov}}{{\mathrm{Oc}}}){\times}{100\%}$$

where Ov is the oviposition rate of mites in the immunized group and Oc is the oviposition rate of mites in the unimmunized group.

The fecundity of mites is the number of eggs produced by mite (F):

4 $${\mathrm{F}} (\%)=\frac{{\text{No. of eggs}}}{\text{No. of mites laying eggs}}{\times}{100\%}$$

Reduction on fecundity of mites (RF):

5 $$\mathrm{RF} (\%)=\left({1-}\frac{\mathrm{Fv}}{{\mathrm{Fc}}}\right){\times}{100\% }$$

where Fv and Fc are the fecundity of mites in the immunized group and the unimmunized group, respectively.

The egg hatching rate (EH):

6 $$\mathrm{EH} (\%)=\frac{\text{No. of larvae}}{\text{No. of eggs}}{\times}{ 100\%}$$

Reduction on egg hatching rate of mites (REH):

7 $$\mathrm{REH} (\%)=\left({1-}\frac{\mathrm{EHv}}{{\mathrm{EHc}}}\right){\times}{100\%}$$

where EHv is the egg hatching rate in the immunized group, and EHc is the egg hatching rate in the unimmunized group.

The digestion rate of mites in digesting period (D):

8 $$\mathrm{D} (\%)=\frac{\text{Average weight after }\text{feeding - Average weight after digesting period}}{\text{Average weight gain after feeding}}{\times}{ 100\%}$$

where D was evaluated on day 3 after collection in our study.

Reduction on digestion rate of mite (DR):

9 $$\mathrm{DR} (\%)=\mathrm{(1-}\frac{\mathrm{DRv}}{{\mathrm{DRc}}}){\times}{100\%}$$

where Dv and Dc are the digestion rate in the immunized group and the unimmunized group, respectively.

We calculated the success rate (S) and vaccine efficacy (E) to describe the efficacy of vaccination better.

10 $$S\left( \% \right) = \left[ {\left( {1 - M} \right) \times O \times F} \right] \times 100\%$$

11 $${\mathrm{E}}  (\%) = \left(1-\frac{{\mathrm{Sv}}}{{\mathrm{Sc}}}\right) \times 100 \%$$

where Sv and Sc are the success rate in the immunized group and the unimmunized group, respectively.

### Quantitative real-time PCR of *Deg-CYP-3* after immunization

To evaluate the impact of vaccine immunization on the mRNA expression of the *Deg-CYP-3* gene in *D. gallinae*, 50 engorged mites from immunized and unimmunized groups were collected, respectively. The expression levels of the *Deg-CYP-3* gene were quantified in two groups via qRT-PCR, following the method described above.

### Statistical analysis

All data are presented as mean ± standard deviation and analyzed using SPSS v26.0 (IBM SPSS Statistics, Armonk, NY, USA). The mRNA expression of *Deg-CYP-3* gene at various developmental stages was analyzed using one-way analysis of variance followed by Tukey’s honestly significant difference test, with significance indicated at *P* < 0.05. The mRNA expression levels in fed and starved mites, antibody response in chicks, the oviposition rate, fecundity, hatching rate, vaccine efficacy, and mortality rate of mites were analyzed using an independent samples *t*-test at a significance level of* P* < 0.05. Graphical representations of the data were generated using GraphPad Prism 9.5 (GraphPad Software, Inc., San Diego, CA, USA).

## Results

### *Deg-CYP-3* expression analysis

**Fig. 1:**
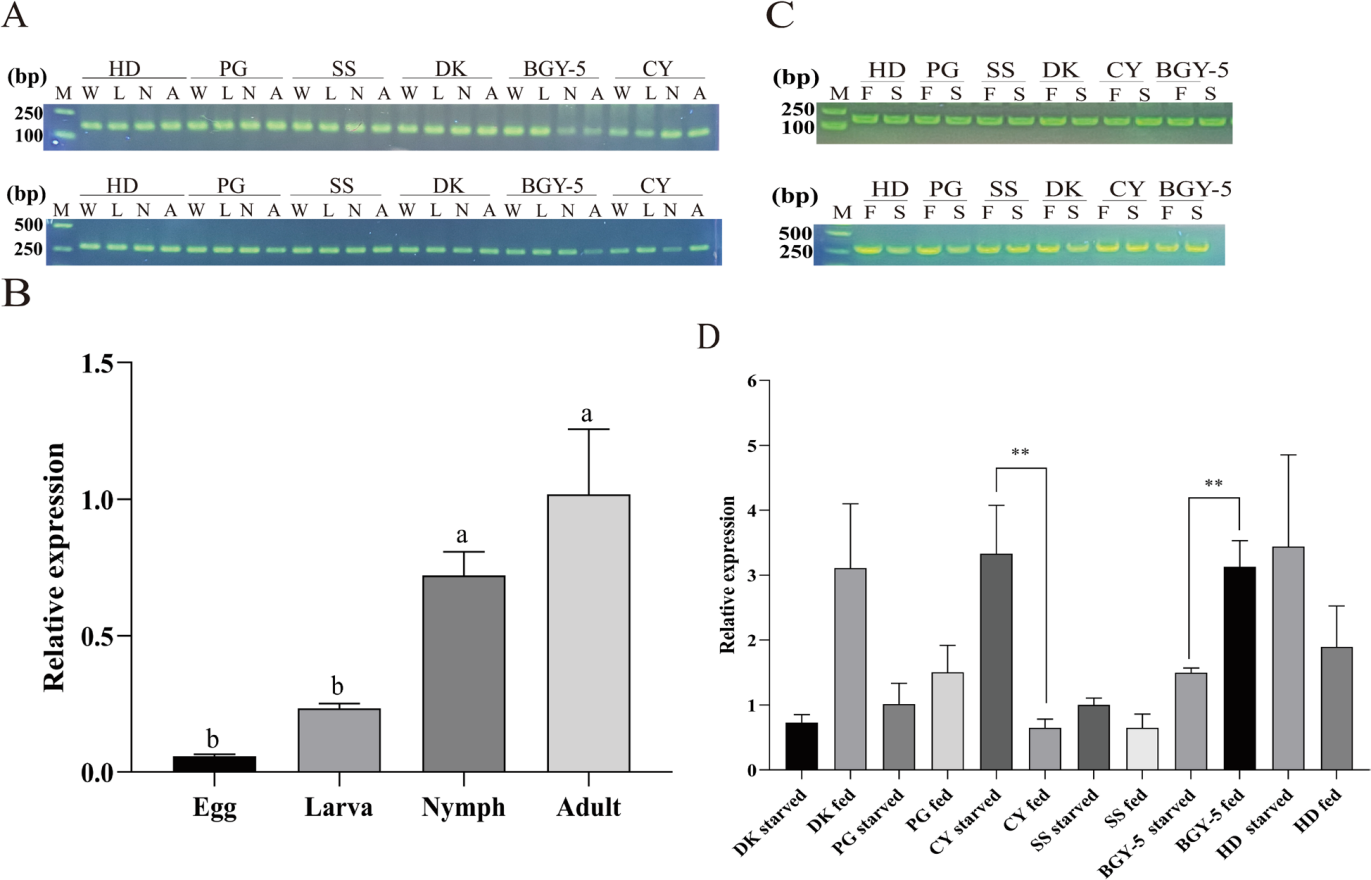
The mRNA expression of *Deg-CYP-3* at different developmental stages as well as under engorged and starved conditions. **A** *Deg-CYP-3* expression at different developmental stages examined by PCR. **B** *Deg-CYP-3* expression at different developmental stages examined by qRT-PCR. **C** *Deg-CYP-3* expression under engorged and starved conditions examined by PCR. **D** *Deg-CYP-3* expression under engorged and starved conditions examined by qRT-PCR. The actin gene was amplified as the internal control. Abbreviations: E: egg; L: larva; N: nymph; A: adult; F: fed; S: starved. Columns labeled with distinct letters are indicated to be significantly different (*P* < 0.05), while asterisks on the error bars are used to denote significant differences in the same strain between the fed and starved mites (*P < 0.05, **P < 0.01)

The mRNA expression profile of the *Deg-CYP-3* gene was analyzed across various developmental stages of *D. gallinae*. A single, distinct target band was consistently detected in all four developmental stages examined (Fig. 1A). To quantify changes in *Deg-CYP-3* gene expression during development in the SS strain, qRT-PCR analysis was performed. As shown in Fig. 1B, relative to eggs, the expression levels increased by 4.0-, 12.3-, and 17.4-fold in larvae, nymphs, and adults, respectively. No significant difference in transcript levels was observed between eggs and larvae (*P* > 0.05); however, expression in both nymphs and adults was significantly higher than in eggs (*P* < 0.05).

To investigate the influence of nutritional status on *Deg-CYP-3* expression, mRNA levels were assessed in adult mites under starved and engorged conditions. PCR results indicated stable expression of the *Deg-CYP-3* gene irrespective of nutritional state (Fig. 1C). Consistent with this, qRT-PCR analysis confirmed that the gene is expressed in adult mites under both fed and starved conditions (Fig. 1D). Notably, differential expression patterns were observed between satiated and starved states across mite strains. Specifically, the DK, PG, and BGY-5 strains exhibited downregulation under starvation, with fold-changes of 4.3-, 1.5-, and 2.9-fold decrease, respectively. In contrast, the CY, SS, and HD strains showed upregulation under starvation, with fold-changes of 5.1-, 1.1-, and 1.8-fold increase, respectively.

### Expression of the recombinant Deg-CYP-3 protein

The 1485 bp PCR amplification product of the *Deg-CYP-3* gene from the SS strain was confirmed to be consistent with the reported sequence through DNA sequencing. In this study, homology and functional analyses were performed on the *Deg-CYP-3* sequences of the SS, DK, and HD strains. The results revealed that the *Deg-CYP-3* sequences across these mite strains (SS, DK, and HD) exhibited a sequence similarity of up to 99.5%, and all contained identical conserved domains, as shown in Supplementary Fig. S1.

**Fig. 2:**
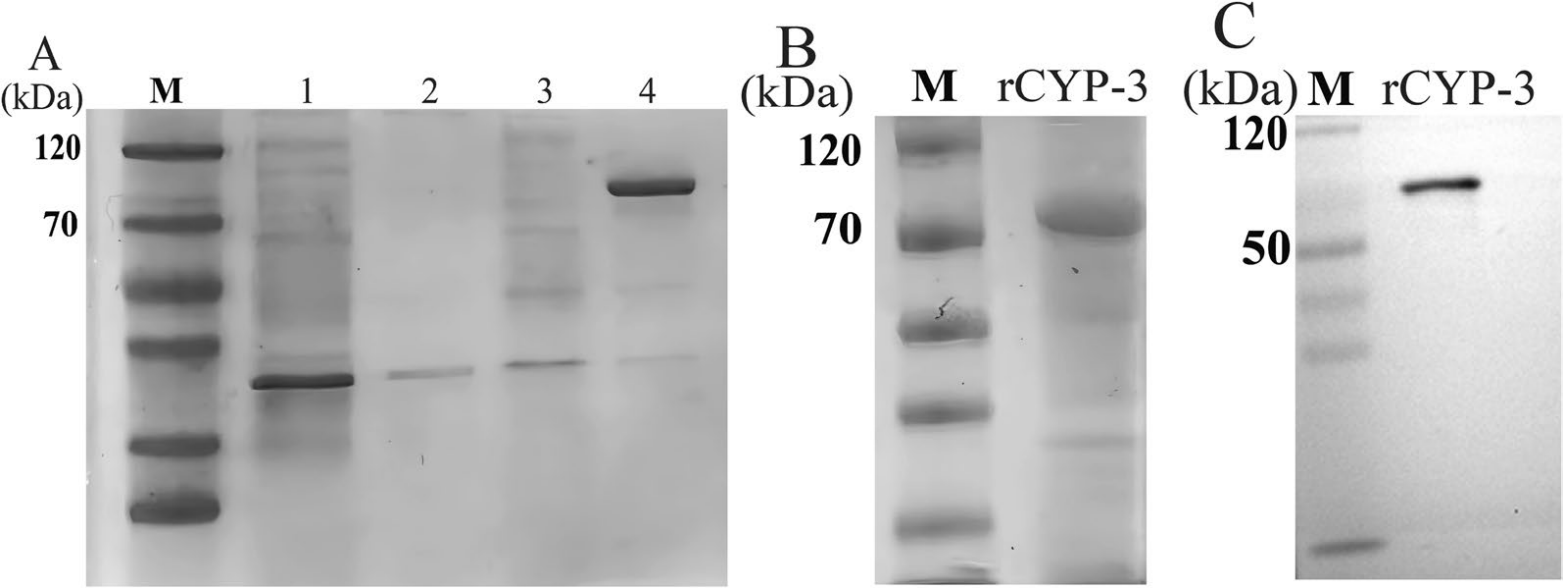
Expression and purification of the recombinant protein rCYP-3. **A** The expression of the recombinant protein rCYP-3 by SDS-PAGE. M: prestained protein ladder, Lane 1: uninduced sediment of Transetta (DE3) cell lysate with pET32a-Deg-CYP-3 vector, Lane 2: uninduced supernatant of Transetta (DE3) cell lysate with pET32a-Deg-CYP-3 vector, Lane 3: induced sediment of Transetta (DE3) cell lysate with pET32a-Deg-CYP-3 vector, Lane 4: induced supernatant of Transetta (DE3) cell lysate with pET32a-Deg-CYP-3 vector. **B** SDS-PAGE analysis of purified rCYP-3. **C** WB analysis of purified rCYP-3

Subsequently, the *Deg-CYP-3* gene was expressed in *E. coli* Transetta (DE3) pLysS using pET-32a as the expression vector. The Deg-CYP-3 protein has a molecular weight of 56.7 kDa, while the vector-derived portion is approximately 20.7 kDa. Thus, the expected size of the recombinant fusion protein was 77.4 kDa. SDS-PAGE analysis revealed a distinct protein band of approximately 77.4 kDa following IPTG induction (Fig. 2A). Affinity chromatography was then used to purify the recombinant protein, and the eluted fraction was analyzed by 10.0% SDS-PAGE. As shown in Fig. 2B, the molecular weight of the purified protein matched the expected size, and the presence of a single band indicated high purity. Finally, western blot analysis using an anti-histidine antibody detected a single positive signal, confirming the expression of the histidine-tagged rCYP-3 protein (Fig. 2C). These findings collectively demonstrate the successful expression and purification of the histidine-tagged rCYP-3 protein in the *E. coli* expression system.

### Analysis of antibody response in immunized chicks by ELISA

**Fig. 3:**
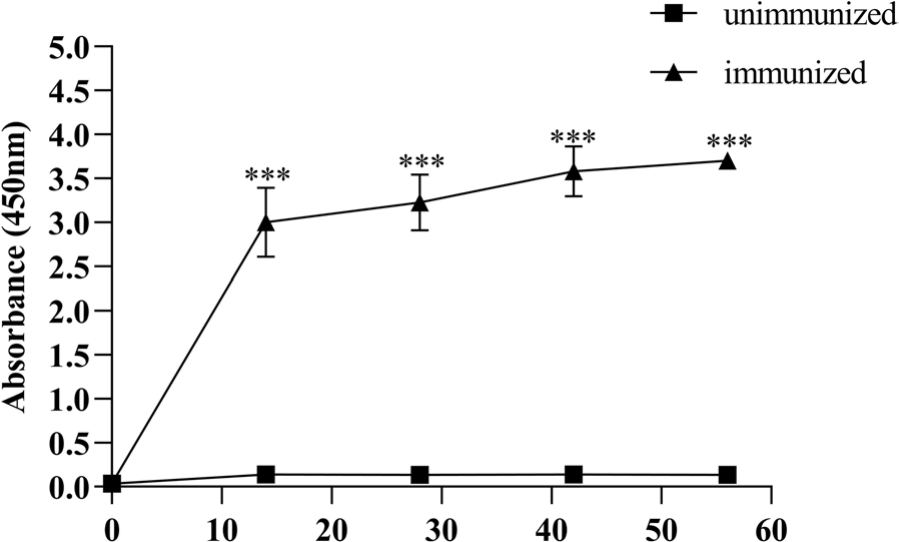
Antibody response of chicks after immunization with rCYP-3. Chicken serum IgY antibody levels were determined by ELISA at OD_450_ nm. The data in the immunized and unimmunized groups were compared by independent-samples Student’s *t*-test (***P < 0.001)

An ELISA was performed to detect antigen-specific IgY immune responses in the sera of immunized chicks (Fig. 3). Over the 56-day observation period, the OD values in the unimmunized group ranged from 0.0 ± 0.0 to 0.1 ± 0.0, whereas those in the immunized group ranged from 0.0 ± 0.0 to 3.7 ± 0.6. Notably, the antibody levels in the immunized group increased sharply after the initial immunization, remained significantly higher than those in the unimmunized group starting at day 14, and persisted at a stable high level throughout the remainder of the observation period (*P* < 0.001).

### Immunization efficacy

**Fig. 4:**
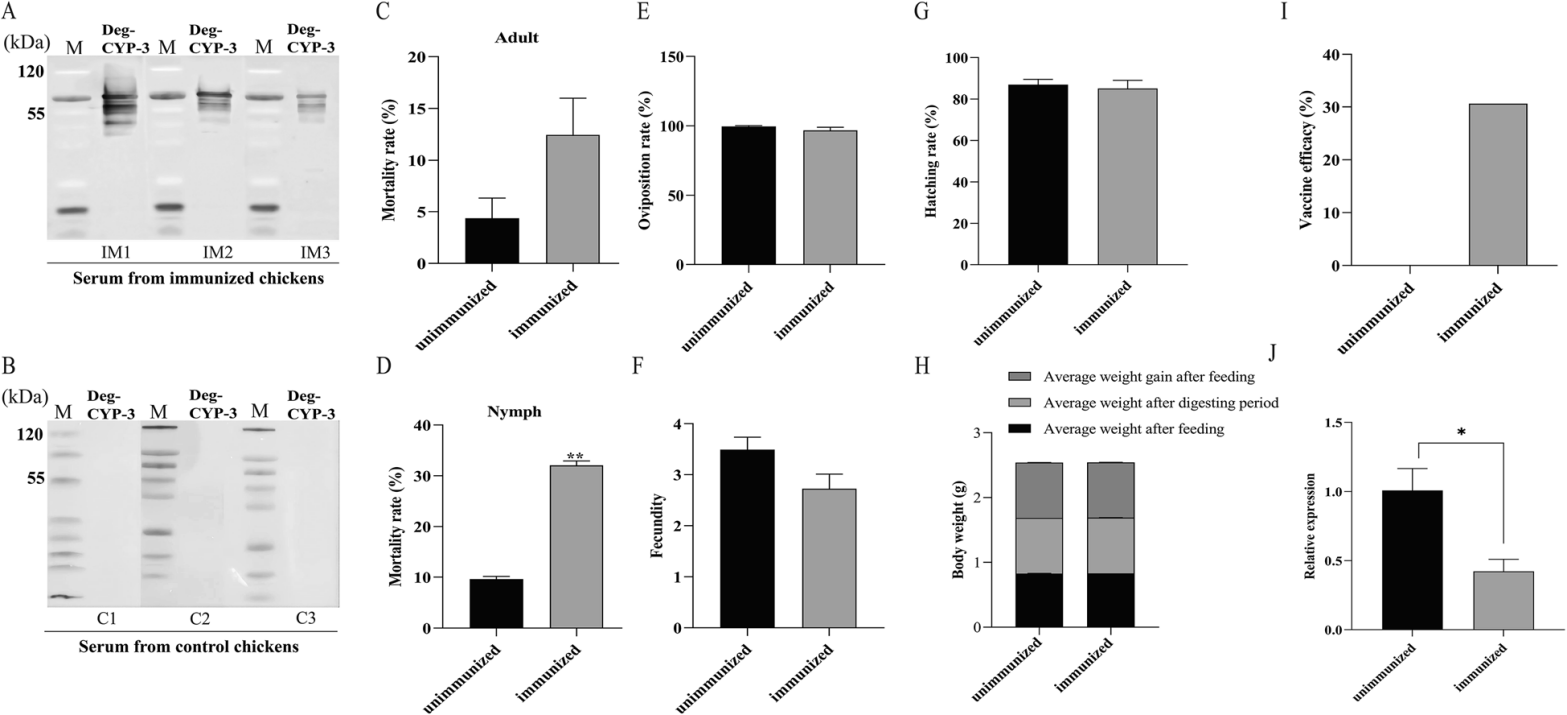
Effects of immunization with rCYP-3 on *D. gallinae*. **A** WB analysis of the reaction between extracted mite protein and immunized serum. **B** WB analysis of the reaction between extracted mite protein and unimmunized serum. **C** Mortality rate of adult. **D** Mortality rate of nymph. **E** Oviposition rate. **F** Fecundity. **G** Hatching rate **H** Body weight. **I** Vaccine efficacy. **J** The relative expression of the *Deg-CYP-3* gene. The results are shown as mean ± SD, and the data were compared by independent-samples Student’s *t*-test between immunized and unimmunized groups (*P < 0.05, **P < 0.01)

To confirm the presence of specific antibodies against the rCYP-3 vaccine antigen in serum, WB analysis was performed. As shown in Fig. 4A, chick anti-rCYP-3 serum specifically recognized the P450s protein (56.7 kDa) extracted from *D. gallinae*. In contrast, sera from unimmunized chicks showed no detectable immunoreactive bands (Fig. 4B), confirming the successful induction of antigen-specific antibody production in immunized chicks. Throughout the experimental period, all chickens remained in good health, exhibiting bright and clean plumage, normal alertness, and consistent activity levels.

To evaluate the potential anti-*D. gallinae* effects of rCYP-3 immunization, various biological parameters were compared between the immunized and unimmunized groups. Adult and nymph mite mortality were analyzed separately. The mortality rate of adult mites in the immunized group reached 12.5% by day 5, higher than the 4.4% observed in the unimmunized group (Fig. 4C); however, this difference was not statistically significant (*P* > 0.05). Notably, nymph mortality in the immunized group reached 32.0% (Fig. 4D), representing a significant increase compared with the 9.6% in the unimmunized group (*P* < 0.01). In addition, nymph mortality was markedly higher than adult mortality, suggesting stage-specific susceptibility.

The effects of immunization on oviposition rate, adult fecundity, hatching rate, blood digestion efficiency, and vaccine efficacy were further evaluated. The results showed that the oviposition rate of female mites in the immunized group was slightly lower than that in the unimmunized group (Fig. 4E), with average oviposition rates of 96.7% and 99.5%, respectively. Similarly, the fecundity of female mites in the immunized group was reduced compared with the unimmunized group (Fig. 4F), with mean egg counts of 2.7 and 3.5, respectively. In addition, the hatching rate in the immunized group was slightly lower than that in the unimmunized group (Fig. 4G), with average values of 85.1% and 87.0%, With respect to body weight, no significant differences were observed between groups in average weight after feeding, average weight after digestion, or average weight gain following feeding (Fig. 4H; *P* > 0.05). Body weight trajectories remained closely aligned across all time points. Vaccine efficacy, calculated on the basis of comparative mite performance, was determined to be 30.6% in the immunized group (Fig. 4I). Collectively, these results support the potential of Deg-CYP-3 as a candidate antigen for a *D. gallinae* vaccine. Furthermore, qRT-PCR analysis revealed a significant downregulation of *Deg-CYP-3* gene expression in the immunized group, with a 2.4-fold reduction compared with the unimmunized group (*P* < 0.05; Fig. 4J). Reproductive parameters and their corresponding percentage reductions are summarized in Table 2.

**Table 2:** The effects of immunization with rCYP-3 on oviposition, fecundity, and hatchability of adult female mites

Experimental group	O (%)	RO (%)	F (eggs/ mite)	RF (%)	EH (%)	REH (%)	M (%)	N (%)	E (%)
Immunized	96.7 ± 2.3	2.8	2.7 ± 0.3	22.0%	85.1 ± 3.9	2.2	12.5 ± 3.5	32.0 ± 0.9^**^	30.6
Unimmunized	99.5 ± 0.7	-	3.5 ± 0.3	–	87.0 ± 2.6	–	4.4 ± 1.9	9.6 ± 0.6	–

O: oviposition rate; RO: oviposition rate decline; F: fecundity; RF: fecundity decline; EH: hatching rate; REH: hatching rate decline; M: mortality rate of adult; N: mortality rate of nymph; E: vaccine efficacy

## Discussion

*Dermanyssus gallinae* regarded as one of the most harmful ectoparasites of the avian species, including chicks. Currently, infestations of *D. gallinae* are typically controlled by the application of synthetic pesticides. However, the resistance of *D. gallinae* to acaricides has been reported recently in many countries. Considering its safety, no chemical residue, prolonged effectiveness, vaccine has shown a promising prospect in the control of *D. gallinae* [10]. The P450 enzymes, as key detoxification enzymes, have been demonstrated to be integral components of biosynthetic and degradative pathways for endogenous compounds (e.g., pheromones, 20-hydroxyecdysone, and juvenile hormone) in insects [29], with significant roles in the growth, development, and reproduction of both insects and mites. In addition, they have been shown to be involved in the detoxification of various exogenous compounds, including pesticides and plant toxins [29]. For example, sustained overexpression of multiple cytochrome P450 genes (CYP9A12, CYP9A14, and CYP6B7) in *H. armigera* has been identified as being linked to pyrethroid resistance [30]. Notably, the P450s play a crucial role not only in insecticide resistance but also display tissue-specific expression patterns. For example, studies on *D. melanogaster* and *H. armigera* [23, 24] have demonstrated that P450s are expressed in the midgut, a result consistent with our observations in *D. gallinae* (unpublished). As a central site for digestion and nutrient absorption in parasites, the gut is frequently exposed to host-ingested blood or food. Proteins expressed in this organ may therefore be directly involved in parasite survival and accessible to the host immune system, making them potential candidates for vaccine development. Consequently, investigating P450s genes as possible vaccine antigens offers promising prospects for managing *D. gallinae* infestations.

The differential expression of CYP genes across life stages and tissues is regarded as a characteristic feature of P450s in insects [23]. In our study, the *Deg-CYP-3* gene was expressed throughout all life stages of *D. gallinae*. Consistent expression patterns were also observed for Deg-CPR-2 and Deg-CPR-1 in *D. gallinae* [15, 31]. These findings also align with previous reports on *Liposcelis bostrychophila* [32] and *M. domestica* [33]. We further found that the *Deg-CYP-3* gene exhibited high transcriptional levels in the adult stage while showing significantly lower expressions in the egg and larval stages. It was reported by Wang et al. that the four P450s genes including *Deg-CYP-3* were highly expressed in the adult stage, while their expressions were markedly lower in the egg and larval stages [23], and this finding is consistent with the results of the current study. Furthermore, it was observed that the stably expressions were present in adult mites, regardless of whether they were in a blood-fed state or a starved state. The P450s can catalyze a number of lipophilic compounds, and are involved in the metabolism of exogenous and endogenous chemical substances. Therefore, we speculate the expression of *Deg-CYP-3* may be upregulated under blood fed conditions to enhance metabolic capacity, as shown in the results of DK, PG, and BGY-5 strains. However, upregulation occurred under starved conditions in other strains, which may be attributed to individual variations.

The expression of heterologous proteins in microorganisms using genetic recombination is still a high point in the development and exploitation of modern biotechnology [34]. To date, reformed *E. coli* has been used extensively as the cellular host for foreign protein expression owing to its rapid growth rate, capacity for continuous fermentation, and relatively low cost [34]. The *E. coli* expression system can efficiently secrete the target protein into the cultural supernatant to reduce endotoxin-related risks, and as a prokaryotic system, it was incapable of post-translational modifications, while it does not affect the biological activity, function, structure, solubility, stability, half-life, protease resistance, or compartmentalization of functional proteins [35]. In the present study, this system was employed for the preparation of rCYP-3 as a vaccine antigen. Notably, the target protein was effectively secreted into the supernatant, as confirmed by WB. It has been reported by Verma et al. that the conversion of zymogens from their inactive form to the active form is influenced both by induction by other enzymes and cofactors, and by a self-catalytic process triggered by changes in pH [36]. In our previous experiments, we found that the rCYP-3 protein exhibited an activity of 0.2 μmol/min/mg toward p-nitroanisole, with optimal pH and temperature of 6.5 and 30 ℃ (unpublished). Recombinant proteins with enzymatic activity may make it easier for animals to produce effective antibodies. In this study, the chicks have been vaccinated four times. The antibody level of the chicks increased dramatically after first immunization, remained at a stable high level from 14 to 56 days. This is consistent with the report by Ariizumi et al., in which elevated antibody titers in immune plasma were observed in all chicks, and the production of Deg-CPR-2-specific antibodies was confirmed via WB [31]. Fujisawa et al. also showed that increased antibody titers were detected in the immune plasmas of chickens, and WB analysis confirmed the presence of antibodies specific to Dg-APMAP-N-his [12]. Our study further confirmed through WB analysis that the produced antibodies included rCYP-3-N-his-specific antibodies, which were consistent with their research findings. It is worth noting that the anti-rCYP-3 serum specifically recognized the P450s proteins extracted from *D. gallinae*. As reported by Wang et al., four P450s genes of *D. gallinae* all contained the conserved motifs, such as the Helix-C, Helix-I, and the meander domain, indicating that they all have conserved functions [21]. Therefore, we speculate that the antibody against Deg-CYP-3 protein may also be able to block the other isoforms of cytochrome P450, thereby inhibiting its function, which needs to be further studied. Similarly, this study found that the Deg-CYP-3 sequences of different mite strains (SS, DK, and HD) shared a sequence similarity of up to 99.5% and all possessed the same conserved domains. Furthermore, field conditions are more likely to result in reduced vaccine persistence. In subsequent studies, it is necessary to further conduct small-scale field trials to continuously monitor the dynamic changes in antibody levels in vaccinated chickens and the effectiveness of mite population control, with the aim of validating and optimizing the long-term protective capacity of this vaccination regimen.

To evaluate the potential of rCYP-3 as a vaccine antigen, we used a vaccine efficacy evaluation method developed by Xu et al. via in vivo rearing system that mimics the natural infestation status of mites to assess the vaccine efficacy against *D. gallinae* [14] Our results showed that the mortality rates of both adults and nymphs increased, with the mortality rate of nymphs rising significantly. However, the mortality rate of adult mites was lower than that of nymphs. Research by Ariizumi et al. [31] demonstrated that Deg-CPR-2 exhibited acaricidal effects on protonymphs but had weak efficacy against adult *D. gallinae*. These findings strikingly analogous to those of the present study. This may be because nymphs are in the early stages of development and their body surface structures (such as the epidermis and body wall) are not yet fully mature, resulting in weaker defenses. Alternatively, the digestive system of nymphs may still be underdeveloped, causing the intestinal epithelial cells to be more vulnerable. In practical clinic application, it is best for the vaccine to be able to kill adult mites, thereby reducing the growth of mite populations. Nevertheless, to a certain extent, killing the nymphs can also lead to a reduction in mite reproduction. In addition to the increase in mite mortality, we also confirmed that immunization with rCYP-3 affected the reproductive capacity of *D. gallinae*. Immunization with rCYP-3 could also reduce mite fecundity, hatching rate, and oviposition rate. However, there was no effect of immunization with rCYP-3 on blood digestion. Overall, the vaccine efficacy of rCYP-3 was 30.6%, and immunization with rCYP-3 effectively reduced mite survival and reproductive capacity, demonstrating its potential as a preventive and control strategy against *D. gallinae*. To date, several candidates for vaccine antigens against *D. gallinae* have been identified [13, 31, 37–39]. The acaricidal rates of cysteine protease and transporter 1 against *D*. *gallinae* were 13.7% and 18.0% on the fifth day, respectively [16, 22]. Similarly, Xu et al. showed that the acaricidal efficacies were 22.2% for rDg-CatD-1, 17.2% for rDg-CatL-1, and 19.5% for rDg-Lgm, and immunization with these antigens controlled *D*. *gallinae* through reduced mite survival and reproductive capacity [14]. The acaricidal efficacy of these vaccines is essentially consistent with that of the vaccine in the present study. However, there is a key distinction: immunization with either rDg-CatD-1 or rDg-CatL-1 can impair blood digestibility, while immunization with rCYP-3 does not have such an effect. This may be due to cathepsin D and cathepsin L, two endopeptidases participating in the digestion of hemoglobin in mites, whereas P450s are not involved in the digestion of hemoglobin in mites. In addition, in comparison with essential oils the acaricidal efficacy of the vaccine investigated in this study is relatively low. Cinnamon has been shown to exhibit an acaricidal efficacy of over 90.0%, while lavender essential oil has also been found to achieve an acaricidal rate exceeding 80.0% [40, 41]. Notably, we found that immunization with rCYP-3 reduced the mRNA expression level of *Deg-CYP-3*. This may be attributed to the fact that the vaccine acts directly on the target gene product, induces RNA degradation, or triggers immune-related transcriptional suppression, with the ultimate consequence that the mRNA expression of the gene in the mites was reduced.

During the trial period, the health status of vaccinated chickens was monitored daily, and no adverse reactions were observed. However, it is widely recognized that immune responses exhibit individual variability. The limited sample size, with only six chickens per group, was deemed insufficient to fully account for such variability. Furthermore, low mite mortality rates were observed with the current vaccine, and the specific reasons for the reduction in mRNA transcription levels following vaccination remain unclear. Moreover, the efficacy of the vaccine under real-world conditions may be constrained. Therefore, to facilitate practical application, dosage optimization and refinement of administration routes should be the focus of subsequent research, with the aim of enhancing the antibody response induced by vaccination. Concurrently, the mechanisms underlying the observed reduction in mRNA transcription levels require further investigation to provide a theoretical basis for improving vaccine efficacy. In addition, accumulating evidence suggests that combining antigens can improve vaccine effectiveness [16, 42, 43]. Fujisawa et al. demonstrated that combining plasma containing antibodies against Dg-Cys with those targeting Dg-Ctr1 or Dg-APMAP can enhance protection against *D. gallinae* [16]. Hence, further investigation is warranted to assess the potential benefits of coadministering this vaccine with others.

## Conclusions

A systematic study was conducted to characterize the *Deg-CYP-3* gene in *D*. *gallinae* and evaluate the immunoprotective efficacy of its corresponding protein vaccine. In *D*. *gallinae*, the *Deg-CYP-3* gene showed high transcriptional activity during both the adult and nymph stages, whereas its expression levels were significantly lower in the egg and larval stages. Moreover, gene expression remains consistent regardless of the feeding status of adult mites, whether they are blood-fed or starved. The recombinant protein rCYP-3 was successfully expressed in *E. coli*. Vaccine efficacy evaluation via in vivo rearing system demonstrated that IgY antibodies generated by chicks against rCYP-3 significantly impacted *D. gallinae* mortality while inhibiting mite oviposition and hatching rates. Noteworthy, the vaccine reduces the expression level of Deg-CYP-3, further demonstrating its efficacy. Taken together, the findings of this study indicate that immunization with rCYP-3 represents a viable strategy to control *D. gallinae* populations in poultry farms.

## Supplementary Information

Additional file1 Figure S1. Alignment of the amino acid sequences of the three strains Deg-CYP-3 gene from *D. gallinae*. The conserved domains common to Deg-CYP-3 gene are boxed. The purple box shows Helix-C (heme-interacting region with typical sequences: WxxxR); the orange box indicates Helix-I (oxygen-binding pocket with the conserved residues: AGxxT); the green box highlights Helix-K (hydrogen-bonding domain: ExxR); the heme-binding domain (FxxGxRxxxG) is boxed in blue

## Data Availability

Data supporting the main conclusions of this study are included in the manuscript.

## Abbreviations

PRM: Poultry red mite
P450s: P450 monooxygenase
SS strain: Relatively susceptible strain
WB: Western Blot
