# Supplementary figures and images for: In vivo evaluation of a cytochrome P450 gene from poultry red mite, Dermanyssus gallinae, as a vaccine antigen for chicks
Source: Parasit Vectors. 2026 Feb 9;19:113. doi: 10.1186/s13071-025-07218-8 (PMC12983623; doi:10.1186/s13071-025-07218-8)

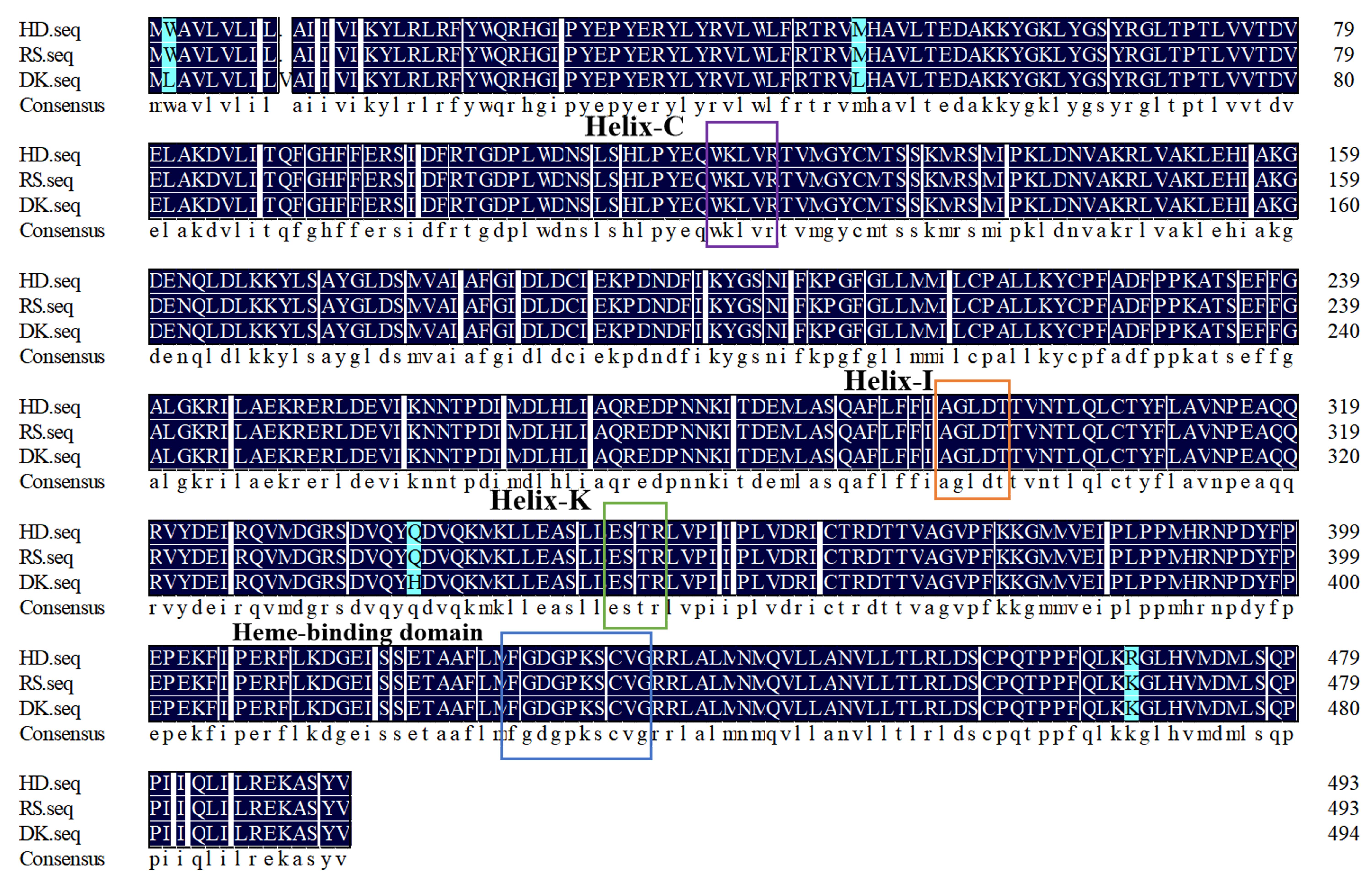

Supplement: Supplementary file 1 — Additional file1 Figure S1. Alignment of the amino acid sequences of the three strains Deg-CYP-3 gene from D. gallinae. The conserved domains common to Deg-CYP-3 gene are boxed. The purple box shows Helix-C (heme-interacting region with typical sequences: WxxxR); the orange box indicates Helix-I (oxygen-binding pocket with the conserved residues: AGxxT); the green box highlights Helix-K (hydrogen-bonding domain: ExxR); the heme-binding domain (FxxGxRxxxG) is boxed in blue [file 13071_2025_7218_MOESM1_ESM.jpg]
